# Association of the non-high-density lipoprotein cholesterol to high-density lipoprotein cholesterol ratio with non-alcoholic fatty liver disease and hepatic steatosis in United States adults: insights from NHANES 2017–2020

**DOI:** 10.3389/fnut.2025.1540903

**Published:** 2025-04-11

**Authors:** Zhen Song, Hai-Qi Gu, Cheng Xu

**Affiliations:** ^**1**^Yancheng Binhai Hospital of Traditional Chinese Medicine, Yancheng, China; ^**2**^Nanjing University of Chinese Medicine, Nanjing, China

**Keywords:** non-alcoholic fatty liver disease, non-high-density lipoprotein cholesterol to high-density lipoprotein cholesterol ratio, hepatic steatosis, liver fibrosis, cross-sectional study, national health and nutrition examination survey

## Abstract

**Objective:**

This study aimed to investigate the association between the non-high-density lipoprotein cholesterol to high-density lipoprotein cholesterol ratio (NHHR) and NAFLD, as well as its relationship with hepatic steatosis and liver fibrosis, in a nationally representative sample of U.S. adults.

**Methods:**

This cross-sectional study analyzed data from 3,529 participants from the National Health and Nutrition Examination Survey in 2017–2020. Multivariable logistic regression and subgroup analyses were used to assess the association between NHHR and NAFLD. Multivariate linear regression was employed to evaluate the relationship between NHHR and hepatic steatosis (controlled attenuation parameter) and liver fibrosis (liver stiffness measurement). Nonlinear relationships were explored through fitted smoothing curves and threshold effect analysis. Receiver operating curve (ROC) analysis was performed to compare the diagnostic performance of NHHR with body mass index (BMI), high-density lipoprotein cholesterol (HDL-C), and total cholesterol (TC).

**Results:**

The study included 3,529 participants (mean age: 51.34 years, 95% CI: 49.97, 52.72), with 53.53% male. NHHR showed a significant positive association with NAFLD after adjusting for confounders (OR: 1.33, 95% CI: 1.24, 1.42). Subgroup analysis indicated a stronger association in females and individuals with normal weight. A nonlinear relationship was identified, with a significant positive association below an inflection point of 4 (OR: 1.52, 95% CI: 1.38, 1.68). NHHR was positively associated with hepatic steatosis but not with liver fibrosis. For NAFLD diagnosis, NHHR achieved an area under the curve (AUC) of 0.66, outperforming TC (AUC = 0.51) but indicating lower accuracy than BMI (AUC = 0.77) and HDL-C (AUC = 0.68).

**Conclusion:**

NHHR is positively associated with NAFLD and hepatic steatosis in U.S. population, highlighting the important role of lipid control in the prevention and clinical management of NAFLD.

## 1 Introduction

Non-alcoholic fatty liver disease (NAFLD) is a prevalent chronic liver condition, affecting approximately one-quarter of the global adult population and posing a significant public health challenge (1, 2). NAFLD covers a spectrum of liver disorders, from non-alcoholic fatty liver to non-alcoholic steatohepatitis, which can progress to advanced liver fibrosis, cirrhosis, and hepatocellular carcinoma (3–5).

Lipid metabolism is pivotal in the pathogenesis of NAFLD, where dyslipidemia serves as a key metabolic hallmark of the disease (6–8). Alterations in lipid profiles are crucial for the assessment and management of NAFLD. The non-high-density lipoprotein cholesterol to high-density lipoprotein cholesterol ratio (NHHR) is an emerging atherogenic lipid indicator, capturing comprehensive data on both atherogenic and anti-atherogenic lipid particles (9). NHHR has proven to be a superior lipid metric for assessing cardiovascular and cerebrovascular disease risk, offering advantages over traditional lipid indicators in evaluating coronary, intracranial atherosclerosis, and arterial stiffness (10–12).

Previous studies in Chinese populations have shown a positive association between higher NHHR and NAFLD (13–15). However, these studies were limited to specific populations and did not explore the relationship between NHHR and the degree of hepatic steatosis and liver fibrosis. This study aims to address this gap by investigating the association between NHHR and the prevalence of NAFLD, and the relationship between NHHR and the severity of hepatic steatosis and liver fibrosis in a nationally representative sample of U.S. adults from the National Health and Nutrition Examination Survey (NHANES).

## 2 Methods

### 2.1 Study design and participants

The NHANES is a cross-sectional health survey in the U.S. that employs a stratified, multistage probability sampling design to obtain a representative sample of the non-institutionalized civilian population (16, 17). All protocols were approved by the National Center for Health Statistics Research Ethics Review Board, and participants provided written informed consent. Detailed information on NHANES is available at https://www.cdc.gov/nchs/nhanes/.

In this study, we used a sample from the 2017–2020 NHANES cycles. Participants aged 20 years or older with complete data on hepatic vibration-controlled transient elastography (VCTE), NHHR, and covariates were included in the analysis. Exclusion criteria were: (1) ineligible, not performed, or partial elastography examination status (*N* = 6,537); (2); hepatitis B (positive for hepatitis B surface antigen) or hepatitis C (positive for hepatitis C antibodies or hepatitis C RNA) infection (*N* = 205); (3) significant alcohol consumption (more than 2 drinks/day for females or 3 drinks/day for males) (*N* = 2,495); (4) use of steatogenic medications (e.g., prednisone, amiodarone, tamoxifen, methotrexate) for at least 3 months (*N* = 15); (5) incomplete NHHR data (*N* = 519); (6) younger than 20 years old (*N* = 1,300); (7) missing covariate data, including education (*N* = 9), marital status (*N* = 4), poverty income ratio (*N* = 616), smoking status (*N* = 1), physical activity (*N* = 5), and the Healthy Eating Index-2015 (*N* = 298). The final study population consisted of 3,529 participants (Supplementary Figure 1).

### 2.2 Assessment of exposure and outcome factors

The NHHR was calculated using participants’ lipid profiles. Non-high-density lipoprotein cholesterol (non-HDL-C) was calculated by subtracting high-density lipoprotein cholesterol (HDL-C) from total cholesterol (TC). NHHR was computed by dividing non-HDL-C by HDL-C. The NHHR formula is: NHHR = Non-HDL-C/HDL-C, where Non-HDL-C = TC - HDL-C.

NAFLD diagnosis traditionally relies on imaging or histological assessment to detect the presence of hepatic steatosis (18). The NHANES staff assessed participants using VCTE with the FibroScan® model 502 V2 Touch. The device measures ultrasound attenuation, recording the controlled attenuation parameter (CAP) as an indicator of hepatic steatosis. We applied a CAP cutoff value of 285 dB/m as the marker for NAFLD status. This cutoff value, validated in the U.S. population, demonstrates 80% sensitivity and 77% specificity for detecting hepatic steatosis (19). Liver fibrosis was assessed using FibroScan®, which employs ultrasound and VCTE to measure liver stiffness. Liver stiffness measurement (LSM) was used to assess the degree of hepatic fibrosis.

### 2.3 Assessment of covariates

To investigate the independent association between NAFLD and NHHR, adjustments were made for potential confounders, including sociodemographic, lifestyle, and health status variables. Sociodemographic factors, such as age, gender, race (Mexican American, Other Hispanic, Non-Hispanic White, Non-Hispanic Black, and Other Races), education level (less than high school, high school, and more than high school), marital status (married/living with partner, widowed/divorced/separated, and never married), poverty income ratio (<1.3, 1.3 to <3.5, and ≥ 3.5) were obtained through interviews. Lifestyle related covariates included smoking status, physical activity, and dietary metric. Smoking status was classified as never smoker former smoker, and current smoker. Physical activity was categorized as inactive, moderate, and vigorous, based on the interview data. Dietary metric was evaluated by the Healthy Eating Index-2015 (HEI-2015), which is a measure for assessing whether a set of foods aligns with the Dietary Guidelines for Americans (20). The components and scoring standards of HEI-2015 were detailed in Supplementary method 1. Body mass index (BMI), and history of hypertension or diabetes were all considered variables of health status. BMI was calculated as weight divided by height squared and categorized as normal weight (<25), overweight (≥25 and < 30), and obesity (≥30) (21). History of hypertension was defined by a prior diagnosis, current use of antihypertensive medications, or a systolic blood pressure of ≥130 mmHg and/or diastolic blood pressure of ≥80 mmHg (22). History of diabetes was defined by a prior diagnosis, current use of insulin or diabetic medications, fasting plasma glucose ≥126 mg/dL, or hemoglobin A1c ≥6.5% (23).

### 2.4 Statistical analysis

The analysis accounted for the complex multistage cluster survey design of NHANES by using appropriate sample weights. Participants were stratified into tertiles according to NHHR levels. Baseline characteristics were summarized using weighted means with 95% confidence intervals (CIs) for continuous variables and weighted percentages (95% CIs) for categorical variables. To compare baseline characteristics across NHHR tertiles, weighted linear regression was used for continuous variables, and chi-squared tests were used for categorical variables. Multivariable logistic regression models assessed the relationship between NAFLD and NHHR, while multivariate linear regression models examined the associations between NHHR and both CAP and LSM (Supplementary method 2). NHHR was modeled as both a continuous variable and a categorical variable (tertiles). Three models were assessed: Model 1 was unadjusted; Model 2 adjusted for gender, age, and race; Model 3 further adjusted for education, marital status, poverty income ratio, BMI, smoking status, physical activity, HEI-2015, hypertension, and diabetes. Results are presented as odds ratios (ORs) or *β* coefficients with 95% CIs. Linear trend tests were used to assess the consistency of relationships. Generalized additive models and smooth curve fittings were employed to explore potential nonlinear associations, adjusting for potential confounders. The inflection point was identified using a recursive algorithm, followed by the application of two-piecewise linear regression models. Likelihood ratio tests were used to compare the one-line linear regression model with the two-piecewise linear regression model.

Subgroup analyses examined the association between NHHR and NAFLD across different age groups, genders, BMI categories, smoking statuses, physical activity levels, and histories of hypertension and diabetes. Interaction tests were employed to assess the consistency of these associations across subgroups.

To evaluate NHHR as a diagnostic marker for NAFLD and compare its diagnostic performance with BMI, TC, and HDL-C, receiver operating characteristic (ROC) curves were constructed, and the area under the curve (AUC) was calculated for each metric. Sensitivity, specificity, and accuracy of the diagnostics were determined. The optimal cutoff value was identified using the Youden index (sensitivity + specificity - 1). AUC values for NHHR and other parameters were compared using the Z test, with Bonferroni correction applied to adjust *p* values for multiple comparisons.

All statistical analyses were performed using R^1^ and EmpowerStats.^2^ Statistical significance was set as two-sided *p* < 0.05.

## 3 Results

### 3.1 Baseline characteristics

The baseline characteristics of the included participants, categorized by tertiles of NHHR, are presented in Table 1. Among the 3,529 participants, the mean age was 51.34 years (95% CI: 49.97, 52.72), with 53.53% being male and 66.25% identified as Non-Hispanic White. The mean NHHR value was 2.83 (95% CI: 2.73, 2.94), and 38.46% of the participants were diagnosed with NAFLD. Compared to those in the lowest tertile of NHHR, participants in the highest tertile were more likely to be male, Non-Hispanic White, less likely to be coupled, have a lower HEI-2015 score, and higher rates of obesity and hypertension. Additionally, they had significantly higher prevalence of NAFLD, as well as higher CAP and LSM values.

**Table 1:** Basic characteristics of the study participants.

Characteristics	NHHR			*p* value
	T1	T2	T3	
Age (years)	52.01 (49.59, 54.43)	52.12 (50.29, 53.95)	49.98 (48.13, 51.83)	0.173
Age group (%)				<0.001
20–39	32.99 (27.11, 39.44)	27.33 (23.20, 31.88)	28.45 (24.10, 33.24)	
40–59	24.15 (19.99, 28.87)	34.64 (29.49, 40.16)	40.65 (36.87, 44.53)	
≥60	42.86 (36.93, 49.00)	38.04 (32.41, 44.00)	30.90 (26.23, 36.00)	
Gender				<0.001
Male	39.91 (33.63, 46.53)	51.70 (47.14, 56.23)	67.92 (63.50, 72.04)	
Female	60.09 (53.47, 66.37)	48.30 (43.77, 52.86)	32.08 (27.96, 36.50)	
Race				<0.001
Mexican American	5.56 (3.79, 8.09)	6.15 (4.31, 8.72)	7.43 (5.06, 10.78)	
Other Hispanic	5.20 (3.58, 7.51)	7.05 (4.78, 10.28)	6.90 (5.28, 8.98)	
Non-Hispanic White	65.51 (58.43, 71.97)	66.32 (60.67, 71.54)	66.85 (59.98, 73.08)	
Non-Hispanic Black	13.95 (9.99, 19.13)	10.45 (7.82, 13.85)	7.26 (5.19, 10.06)	
Other Race	9.78 (7.10, 13.31)	10.03 (7.57, 13.17)	11.55 (8.48, 15.56)	
Education				0.058
Less than high school	9.02 (7.56, 10.72)	9.20 (7.31, 11.52)	11.18 (9.41, 13.24)	
High school	22.29 (18.48, 26.64)	24.36 (19.57, 29.88)	27.24 (22.04, 33.16)	
More than high school	68.68 (64.21, 72.83)	66.44 (60.43, 71.96)	61.57 (55.46, 67.34)	
Marital status				0.014
Married/living with partner	19.70 (14.75, 25.81)	15.19 (11.83, 19.30)	13.54 (9.90, 18.25)	
Widowed/divorced/separated	21.04 (17.06, 25.65)	20.98 (17.56, 24.87)	15.36 (12.58, 18.61)	
Never married	59.26 (54.07, 64.24)	63.82 (59.49, 67.94)	71.10 (65.23, 76.34)	
Poverty income ratio				0.622
<1.3	14.29 (11.75, 17.26)	17.43 (15.16, 19.96)	17.05 (14.05, 20.55)	
1.3 to <3.5	36.20 (30.66, 42.14)	34.53 (29.76, 39.63)	33.08 (27.93, 38.67)	
≥3.5	49.51 (42.30, 56.74)	48.04 (42.18, 53.95)	49.87 (42.73, 57.01)	
BMI				<0.001
Normal weight (<25)	41.56 (37.40, 45.84)	24.60 (21.23, 28.31)	10.26 (7.89, 13.22)	
Overweight (25 to <30)	30.44 (27.27, 33.81)	35.76 (32.06, 39.63)	34.24 (27.36, 41.87)	
Obesity (≥30)	28.00 (24.32, 31.99)	39.64 (35.90, 43.51)	55.50 (48.59, 62.21)	
Smoking status				0.183
Never smoker	66.71 (61.05, 71.93)	64.72 (59.61, 69.52)	60.31 (54.82, 65.55)	
Former smoker	25.15 (20.21, 30.84)	24.59 (21.47, 28.00)	29.94 (24.82, 35.61)	
Current smoker	8.14 (5.86, 11.18)	10.69 (8.26, 13.71)	9.75 (7.67, 12.32)	
Physical activity				0.521
Inactive	47.96 (42.52, 53.44)	49.30 (44.91, 53.71)	47.03 (42.28, 51.84)	
Moderate	29.53 (25.35, 34.08)	26.16 (22.35, 30.37)	26.14 (22.03, 30.72)	
Vigorous	22.51 (17.46, 28.51)	24.53 (20.80, 28.70)	26.83 (23.07, 30.95)	
HEI-2015	52.78 (51.24, 54.31)	49.28 (47.98, 50.59)	48.61 (47.71, 49.52)	<0.001
Hypertension (%)				<0.001
No	54.56 (49.53, 59.50)	46.62 (42.37, 50.92)	39.84 (34.01, 45.98)	
Yes	45.44 (40.50, 50.47)	53.38 (49.08, 57.63)	60.16 (54.02, 65.99)	
Diabetes (%)				0.055
No	83.94 (80.66, 86.75)	81.75 (78.84, 84.35)	78.30 (74.04, 82.04)	
Yes	16.06 (13.25, 19.34)	18.25 (15.65, 21.16)	21.70 (17.96, 25.96)	
HDL (mmol/L)	1.67 (1.62, 1.72)	1.34 (1.31, 1.36)	1.07 (1.05, 1.09)	<0.001
TC (mmol/L)	4.28 (4.17, 4.39)	4.76 (4.67, 4.86)	5.45 (5.38, 5.53)	<0.001
NAFLD				<0.001
No	76.99 (73.77, 79.93)	62.63 (57.61, 67.40)	46.15 (41.29, 51.08)	
Yes	23.01 (20.07, 26.23)	37.37 (32.60, 42.39)	53.85 (48.92, 58.71)	
CAP (dB/m)	239.35 (234.67, 244.03)	264.48 (259.05, 269.92)	291.77 (286.20, 297.34)	<0.001
LSM (kPa)	5.57 (5.17, 5.98)	5.49 (5.21, 5.77)	6.22 (5.71, 6.73)	0.034

Weighted mean (95% CI) for continuous variables: *p* value was calculated by weighted linear regression; Weighted percentages (95% CI) for category variables: *p* value was calculated by weighted Chi-square test. A *p* value < 0.05 indicates statistical difference between groups. BMI, body mass index; CAP, controlled attenuation parameter; HEI-2015, Healthy Eating Index-2015; LSM, liver stiffness measurement; NAFLD, non-alcoholic fatty liver disease; NHHR, non-high-density lipoprotein cholesterol to high-density lipoprotein cholesterol ratio; T, tertile.

### 3.2 Association between NHHR and NAFLD

**Figure 1:**
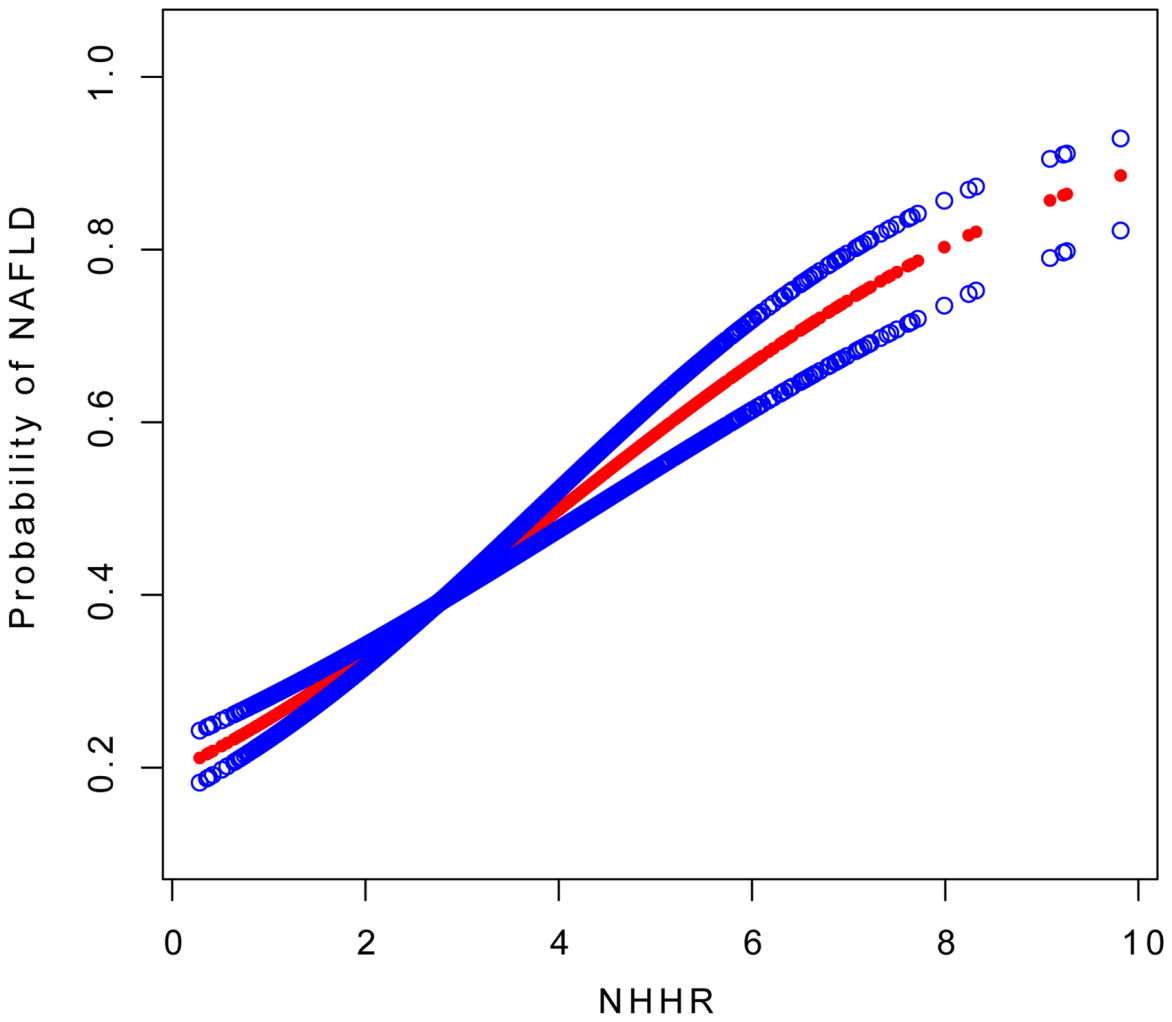
The association between NHHR and NAFLD. The solid red line represents the smooth curve fitting between variables, with blue bands representing the 95% CI of the fitting. NAFLD, non-alcoholic fatty liver disease; NHHR, non-high-density lipoprotein cholesterol to high-density lipoprotein cholesterol ratio.

The multivariate logistic regression analysis revealed a significant association between NAFLD and NHHR across all models (Table 2). NHHR was significantly associated with NAFLD in the non-adjusted model (OR: 1.56, 95% CI: 1.47, 1.66), the partially adjusted model (OR: 1.54, 95% CI: 1.45, 1.64), and the fully adjusted model (OR: 1.33, 95% CI: 1.24, 1.42). NHHR was further categorized into tertiles for sensitivity analysis. In the fully adjusted model, compared to the lowest tertile of NHHR, the probability of NAFLD was increased in both the second tertile (OR: 1.42,95% CI: 1.16, 1.75) and the third tertile (OR = 2.35, 95% CI: 1.91, 2.90), with a significant linear trend (*p* for trend <0.001). To assess potential nonlinearity, we employed smooth curve fitting, which revealed a segmented relationship between NHHR and NAFLD probability (Figure 1). An inflection point was identified at an NHHR of 4. Below this threshold, NHHR showed a significant positive association with NAFLD (OR: 1.52, 95% CI: 1.38, 1.68), whereas above the threshold, the association was not significant (OR: 1.03, 95% CI: 0.91, 1.16) (Table 3).

**Table 2:** Associations between NHHR and NAFLD.

	Model 1^a^		Model 2^b^		Model 3^c^	
	OR (95% CI)	*p* value	OR (95% CI)	*p* value	OR (95% CI)	*p* value
Continuous	1.56 (1.47, 1.66)	<0.001	1.54 (1.45, 1.64)	<0.001	1.33 (1.24, 1.42)	<0.001
Categories						
T1	Reference		Reference		Reference	
T2	1.80 (1.50, 2.15)	<0.001	1.75 (1.46, 2.10)	<0.001	1.42 (1.16, 1.75)	<0.001
T3	3.79 (3.18, 4.52)	<0.001	3.65 (3.04, 4.39)	<0.001	2.35 (1.91, 2.90)	<0.001
*p* for trend		<0.001		<0.001		<0.001

Results are presented as OR (95% CI) and *p* value, with *p* value < 0.05 indicating the statistical significance of the association. NHHR is analyzed both as a continuous variable to assess the linear relationship and as a categorical variable (tertiles) to explore the trend. *p* values for trend tests indicates the significance of the linear trend across tertiles, with *p* for trend value < 0.05 indicating a significant trend. ^a^Model 1: adjusted for no covariates. ^b^Model 2: adjusted for age, gender, and race. ^c^Model 3: adjusted for age, gender, race, education, marital status, poverty income ratio, BMI, smoking status, physical activity, HEI-2015, hypertension, and diabetes. CI, confidence interval; NAFLD, non-alcoholic fatty liver disease; NHHR, non-high-density lipoprotein cholesterol to high-density lipoprotein cholesterol ratio; OR, odds ratio; T, tertile.

**Table 3:** Threshold effect analysis of NHHR on NAFLD.

Outcome	OR (95% CI)	*p* value
One - line linear regression model ^a^	1.33 (1.24, 1.42)	<0.001
Two - piecewise linear regression model ^b^		
NHHR≤4	1.52 (1.38, 1.68)	<0.001
NHHR>4	1.03 (0.91, 1.16)	0.633
Log - likelihood ratio test ^c^		<0.001

Results are presented as OR (95% CI) and *p* value, with *p* value < 0.05 indicating the statistical significance of the association. ^a^One - line linear regression model assesses the association between NHHR and NAFLD using a single linear relationship across the entire range of NHHR values. ^b^Two - piecewise linear regression model assesses the association between NHHR and NAFLD with a potential threshold effect at a NHHR value of 4. The relationship is analyzed separately for NHHR values ≤ 4 and > 4. ^c^Log - likelihood ratio test compares the one-line linear regression model with the two-piecewise linear regression model to determine if the two-piecewise linear regression model significantly improves the fit. A *p* value <0.05 indicates that the two-piecewise linear regression model provides a significantly better fit to the data compared to the one-line linear regression model. CI, confidence interval; NAFLD, non-alcoholic fatty liver disease; NHHR, non-high-density lipoprotein cholesterol to high-density lipoprotein cholesterol ratio; OR, odds ratio.

### 3.3 Association between NHHR and CAP

NHHR showed a significant positive association with CAP across all models, including the fully adjusted model (*β*: 7.41, 95% CI: 6.07, 8.75) (Supplementary Table 1). When categorized into tertiles, participants in the highest NHHR tertile had significantly higher CAP values compared to those in the lowest tertile (*β*: 25.83, 95% CI: 21.51, 30.16), with a significant linear trend (*p* for trend <0.001). To investigate potential nonlinear patterns, we applied smooth curve fitting, which identified a segmented relationship between NHHR and CAP (Supplementary Figure 2). The inflection point was observed at an NHHR of 4. Below this value, NHHR was significantly associated with CAP (*β*: 12.66, 95% CI: 10.65, 14.67), whereas above this value, the association was not significant (*β*: -0.18, 95% CI: −2.73, 2.38) (Supplementary Table 3).

### 3.4 Association between NHHR and LSM

In contrast to the findings for CAP, NHHR was not significantly associated with LSM in the fully adjusted model (β: 0.00, 95% CI: −0.13, 0.12) (Supplementary Table 2). Comparison across NHHR tertiles revealed no significant differences between the highest and lowest tertiles (β: -0.03, 95% CI: −0.44, 0.37), and no significant trend (*p* for trend = 0.866). To explore potential nonlinear relationships, we utilized smooth curve fitting (Supplementary Figure 3). The log-likelihood ratio test indicated that a two-piecewise linear regression model did not provide a better fit than a one-line linear regression model (*p* = 0.128) (Supplementary Table 4).

### 3.5 Subgroup analysis

**Figure 2:**
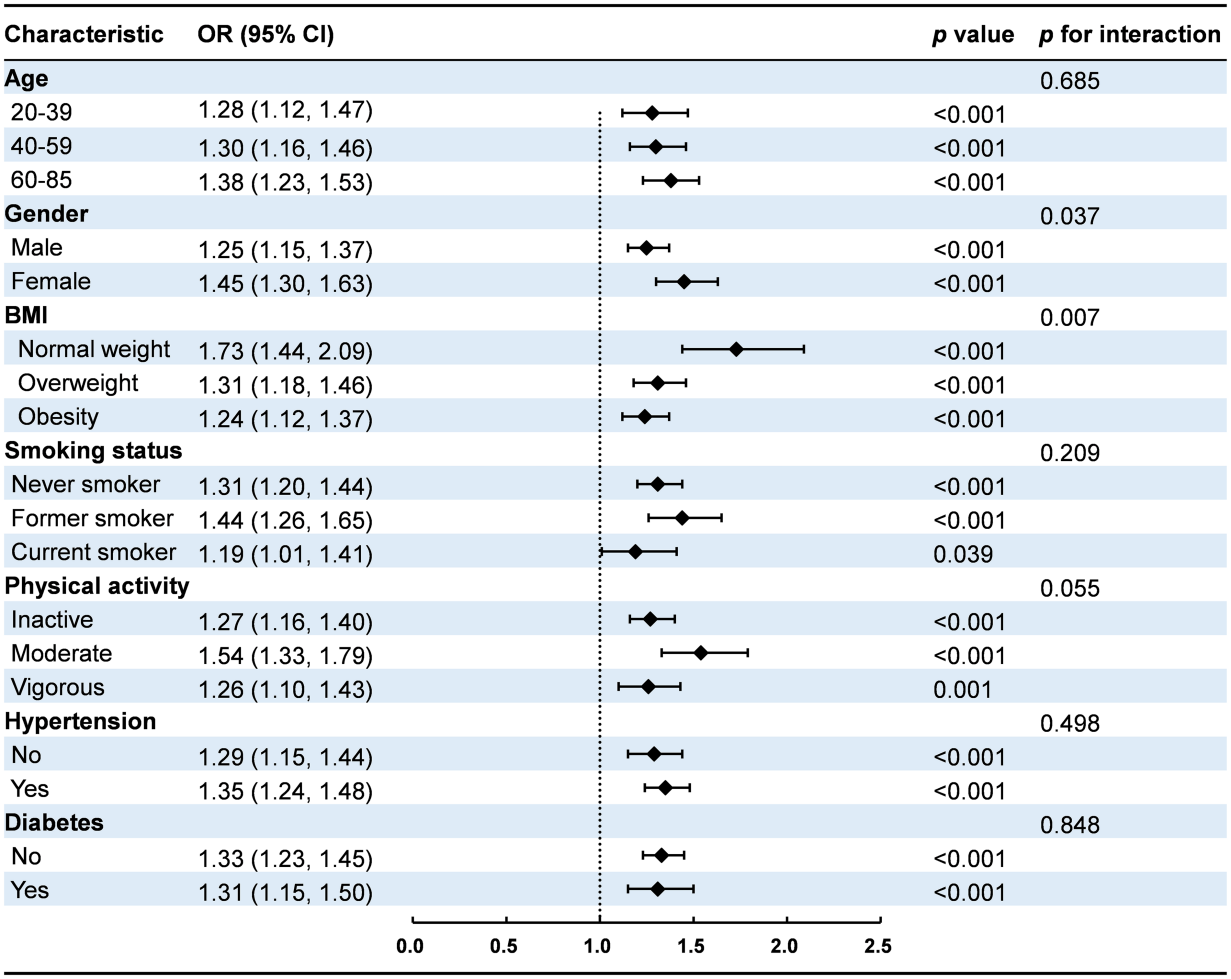
Subgroup analysis for the association between NHHR and NAFLD. Results are presented as OR (95% CI) and *p* value, with *p* < 0.05 indicating statistical significance. *p* for interaction values assess whether the association between NHHR and NAFLD significantly differs within subgroups, with *p* for interaction <0.05 indicating significant interaction effects. BMI, body mass index; CI, confidence interval.

Subgroup analyses, as illustrated in Figure 2, indicated a positive association between NHHR and NAFLD across all subgroups. Significant interactions were identified between NHHR and both gender and BMI categories (*p* for interaction <0.05). The positive association between NHHR and NAFLD was stronger in females (OR: 1.45, 95% CI: 1.30, 1.63) than in males (OR: 1.25, 95% CI: 1.15, 1.37). The NHHR and BMI interaction revealed the strongest association in individuals with normal weight (OR: 1.73, 95% CI: 1.44, 2.09), followed by those who were overweight (OR: 1.31, 95% CI: 1.18, 1.46), and those with obesity (OR: 1.24, 95% CI: 1.12, 1.37). Other factors, including age, smoking status, physical activity, and history of hypertension and diabetes, did not significantly influence the association between NHHR and NAFLD (*p* for interaction >0.05).

### 3.6 Diagnostic performance

**Figure 3:**
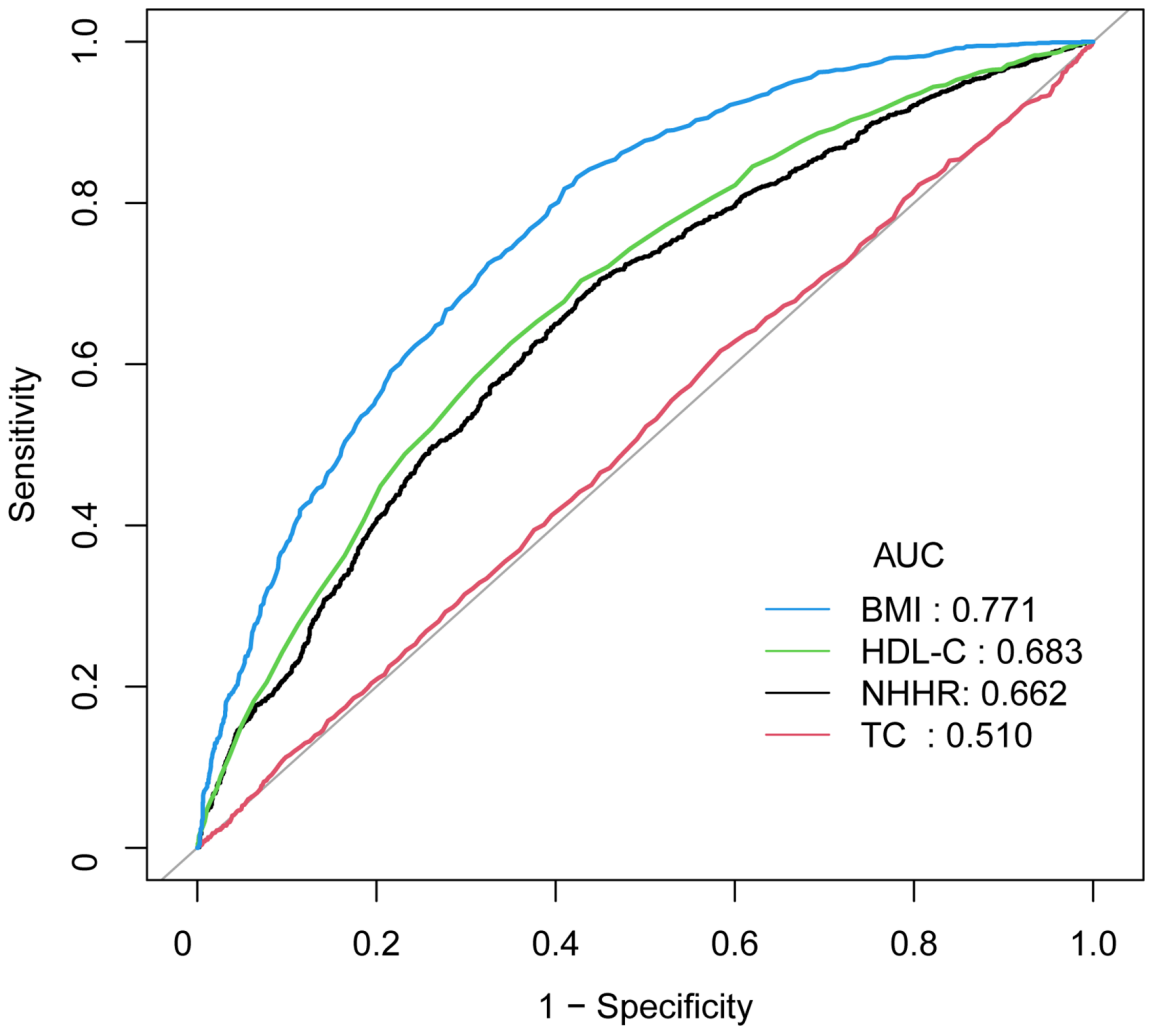
ROC Curves for diagnostic evaluation of NAFLD. AUC, area under the curve; BMI, body mass index; HDL-C, high-density lipoprotein cholesterol; NHHR, non-high-density lipoprotein cholesterol to high-density lipoprotein cholesterol ratio; TC, total cholesterol.

ROC curve analyses were conducted to evaluate the diagnostic value of NHHR for NAFLD compared with other measures (Figure 3). As shown in Table 4, with a cutoff value of 2.40, NHHR achieved a sensitivity of 0.71 and a specificity of 0.55, resulting in an AUC of 0.66 (95% CI: 0.64, 0.68). NHHR outperformed TC, which had an AUC of 0.51 (*p* < 0.001). However, the diagnostic accuracy of NHHR was lower than that of HDL-C, which had an AUC of 0.68 (*p* = 0.015), and BMI, which showed the highest diagnostic efficacy with an AUC of 0.77 (*p* < 0.001).

**Table 4:** Comparison of diagnostic accuracy for NAFLD.

Test	Cut-off value	Sensitivity	Specificity	AUC (95% CI)	*p* value (Adjusted)
NHHR	2.40	0.71	0.55	0.66 (0.64, 0.68)	Reference
TC	4.49	0.62	0.42	0.51 (0.49, 0.53)	<0.001
HDL-C	1.26	0.63	0.65	0.68 (0.67, 0.70)	0.015
BMI	27.45	0.82	0.59	0.77 (0.76, 0.79)	<0.001

*p values have been adjusted using the Bonferroni correction to account for multiple comparisons. A p value <0.05 indicates statistical significance. AUC, area under the curve; BMI, body mass index; CI, confidence interval; HDL-C, high-density lipoprotein cholesterol; NAFLD, non-alcoholic fatty liver disease; NHHR, non-high-density lipoprotein cholesterol to high-density lipoprotein cholesterol ratio; TC, total cholesterol.*

## 4 Discussion

In this nationally representative cross-sectional study, we found a positive association between NHHR and NAFLD in U.S. adults. The analysis revealed a segmented relationship between NHHR and NAFLD, with a threshold effect at an NHHR of 4. Below this threshold, NHHR showed a significant positive association with NAFLD, whereas above this threshold, the association was not significant. ROC curve analysis indicated that NHHR achieved an AUC of 0.66 in diagnosing NAFLD, although it was less effective compared to BMI and HDL-C.

Dysregulation of cholesterol homeostasis is a key metabolic factor in NAFLD pathogenesis (24, 25). Accumulating evidence showed that abnormality in the lipid profile was significantly associated with an increased risk of NAFLD in the general population (26–29). Our study in a U.S. population confirms the positive association between NHHR and NAFLD, consistent with previous findings in Chinese populations (13–15). Additionally, we observed that NHHR was positively correlated with hepatic steatosis but showed no significant relationship with liver fibrosis.

Subgroup analysis revealed significant interactions between NHHR and both gender and BMI. Gender differences exist in the development of NAFLD. These differences are related to sex hormones, particularly estrogen (30, 31). Previous research has demonstrated that the association between dyslipidemia and NAFLD is stronger in females than in males, which supports our findings (32, 33). Furthermore, the association between NHHR and NAFLD was strongest in individuals with normal weight in this study. This finding highlights the importance of monitoring lipid profiles in non-obese individuals who may still be at risk for NAFLD (34, 35).

Obesity and lipid profile parameters are effective markers for predicting NAFLD and are widely used in epidemiological studies (36). Consistent with previous research (13), our findings indicate that NHHR can serve as a valuable marker for NAFLD, demonstrating a fair predictive ability (AUC = 0.66). However, BMI showed superior predictive performance, with an AUC of 0.77. Nonetheless, NHHR still offers clinical value in NAFLD prediction, particularly due to its association with lipid metabolism and dyslipidemia-related disorders. Future studies could enhance the diagnostic accuracy of NAFLD by employing machine learning and integrating a broader range of indicators (37).

In the pathogenesis of NAFLD, hepatic lipid accumulation arises from disruptions in several metabolic pathways, including inadequate uptake of circulating lipids, increased *de novo* lipogenesis, insufficient fatty acid oxidation, and altered export of lipids as components of very low-density lipoprotein (38, 39). Dysregulated lipid metabolism fosters a pro-atherogenic environment, characterized by hypertriglyceridemia, the accumulation of triglyceride-rich lipoproteins, an increase in small, dense low-density lipoprotein cholesterol particles, and reduced HDL-C levels, creating an imbalance between protective and atherogenic lipoproteins (40, 41). This lipid imbalance may contribute to the development of hepatic steatosis through increased lipid peroxidation and oxidative stress (42). The accumulation of cholesterol in hepatocytes induces lipotoxicity, which triggers cellular damage, inflammation, and fibrosis, further exacerbating liver disease severity (43). Atherogenic dyslipidemia, closely linked to disrupted cholesterol metabolism within hepatocytes, not only exacerbates hepatic injury but is also strongly associated with an increased risk of cardiovascular disease in NAFLD patients (8, 44, 45). Moreover, genetic variations, such as mutations in the PCSK7 gene, have been associated with dyslipidemia and more severe liver disease in NAFLD (46). Further exploration of the relationship between NHHR and the pathogenesis of NAFLD would help better understand its role in the disease and promote the development of personalized treatment.

The main strengths of this study include the use of a large, nationally representative sample of U.S. adults, which allows the generalization of findings to a broader population. However, several limitations should be acknowledged. Firstly, the cross-sectional design of the study limits our ability to establish a causal relationship between NHHR and NAFLD. Secondly, the diagnosis of NAFLD was based on VCTE results. While VCTE provides valuable insights into hepatic steatosis and fibrosis, it does not fully match the diagnostic precision of liver biopsy. Thirdly, despite our efforts to adjust for potential confounding factors, unaccounted confounders, such as genetic predispositions and environmental influences, may still affect the observed associations between NHHR and NAFLD. Finally, this study focused exclusively on the U.S. population, which may limit the generalizability of the findings to other ethnic or geographic groups.

## 5 Conclusion

Our study revealed that NHHR is positively associated with NAFLD and hepatic steatosis in U.S. population. Our findings highlight the importance of integrating lipid management into the prevention and clinical management of NAFLD.

## Data Availability

The original contributions presented in the study are included in the article/Supplementary material, further inquiries can be directed to the corresponding author.

## Footnotes

^1^http://www.r-project.org; version 4.4.0

^2^http://www.empowerstats.com; version 4.2
