# Supplementary material for: Association of the non-high-density lipoprotein cholesterol to high-density lipoprotein cholesterol ratio with non-alcoholic fatty liver disease and hepatic steatosis in United States adults: insights from NHANES 2017–2020
Source: Front Nutr. 2025 Apr 11;12:1540903. doi: 10.3389/fnut.2025.1540903 (PMC12021641; doi:10.3389/fnut.2025.1540903)
Supplement: Supplementary file 1 [file Presentation_1.pdf]

Association of the non-high-density lipoprotein cholesterol to high-density lipoprotein cholesterol ratio (NHHR) with non-alcoholic fatty liver disease and hepatic steatosis in United States adults: insights from NHANES 2017-2020

Supplementary Material

Supplementary Method 1 The components and scoring standards of HEI-2015

Supplementary Method 2 Formulas of statistical models

Supplementary Table 1 Associations between NHHR and CAP

Supplementary Table 2 Associations between NHHR and LSM

Supplementary Table 3 Threshold effect analysis of NHHR on CAP

Supplementary Table 4 Threshold effect analysis of NHHR on LSM

Supplementary Figure 1 Flow chart of participants selection

Supplementary Figure 2 The association between NHHR and CAP

Supplementary Figure 3 The association between NHHR and LSM

Supplementary Method 1 The components and scoring standards of HEI-2015

The Healthy Eating Index-2015 (HEI-2015) is a measure for assessing dietary quality, precisely, the degree to which a set of foods aligns with the Dietary Guidelines for Americans. Adequacy components represent the food groups, subgroups, and dietary elements that are encouraged. For these components, higher scores reflect higher intakes, because higher intakes are desirable. Moderation components represent the food groups and dietary elements for which there are recommended limits to consumption. For moderation components, higher scores reflect lower intakes, because lower intakes are more desirable. Intakes between the standard for maximum score and standard for maximum score are scored proportionately. HEI-2015 scores ranged from 0–100, with higher HEI scores reflecting better diet quality. The components and scoring standards of HEI-2015 are as follows (1).

Component	Maximum points	Standard for maximum score	Standard for minimum score of zero
**Adequacy**			
Total Fruits [a]	5	≥0.8 cup equiv. per 1,000 kcal	No Fruit
Whole Fruits [b]	5	≥0.4 cup equiv. per 1,000 kcal	No Whole Fruit
Total Vegetables [c]	5	≥1.1 cup equiv. per 1,000 kcal	No Vegetables
Greens and Beans [c]	5	≥0.2 cup equiv. per 1,000 kcal	No Dark Green Vegetables or Legumes
Whole Grains	10	≥1.5 oz equiv. per 1,000 kcal	No Whole Grains
Dairy [d]	10	≥1.3 cup equiv. per 1,000 kcal	No Dairy
Total Protein Foods [c]	5	≥2.5 oz equiv. per 1,000 kcal	No Protein Foods

Seafood [e] and Plant Proteins [c]	5	≥0.8 oz equiv. per 1,000 kcal	No Seafood or Plant Proteins
Fatty Acids [f]	10	(PUFAs + MUFAs)/SFAs ≥2.5	(PUFAs + MUFAs)/SFAs ≤1.2
**Moderation**			
Refined Grains	10	≤1.8 oz equiv. per 1,000 kcal	≥4.3 oz equiv. per 1,000 kcal
Sodium	10	≤1.1 gram per 1,000 kcal	≥2.0 grams per 1,000 kcal
Added Sugars	10	≤6.5% of energy	≥26% of energy
Saturated Fats	10	≤8% of energy	≥16% of energy

[a] Includes 100% fruit juice.

[b] Includes all forms except juice.

[c] Includes legumes (beans and peas).

[d] Includes all milk products, such as fluid milk, yogurt, and cheese, and fortified soy beverages.

[e] Includes seafood, nuts, seeds, soy products (other than beverages), and legumes (beans and peas).

[f] Ratio of poly- and monounsaturated fatty acids (PUFAs and MUFAs) to saturated fatty acids (SFAs).

Abbreviations: equiv., equivalents; PUFAs, polyunsaturated fatty acids; bMUFAs, monounsaturated fatty acids; cSFAs, saturated fatty acids.

Reference

1. Krebs-Smith SM, Pannucci TE, Subar AF, Kirkpatrick SI, Lerman JL, Tooze JA, et al. Update of the Healthy Eating Index: HEI-2015. J Acad Nutr Diet. 2018;118(9):1591-602

Supplementary Method 2 Formulas of statistical models

In this study, the following statistical models were used to assess the associations between NHHR and NAFLD, CAP, and LSM. The formulas for each model are presented below, along with a description of the variables included.

1. **Logistic regression model for NAFLD**

Formula: $\text{logit}(P(\text{NAFLD})) = \beta_0 + \beta_1(\text{NHHR}) + \beta_2(\text{covariate}_1) + \ldots + \beta_n(\text{covariate}_n)$

Dependent Variable: P(NAFLD): Probability of NAFLD (binary outcome: yes/no).

Independent Variable: NHHR.

Covariates: age, gender, race, education, marital status, poverty income ratio, BMI, smoking status, physical activity, HEI-2015, hypertension, and diabetes.

2. **Linear regression model for CAP**

Formula: $\text{CAP} = \beta_0 + \beta_1(\text{NHHR}) + \beta_2(\text{covariate}_1) + \ldots + \beta_n(\text{covariate}_n)$

Dependent Variable: CAP.

Independent Variable: NHHR.

Covariates: age, gender, race, education, marital status, poverty income ratio, BMI, smoking status, physical activity, HEI-2015, hypertension, and diabetes.

3. **Linear regression model for LSM**

Formula: $\text{LSM} = \beta_0 + \beta_1(\text{NHHR}) + \beta_2(\text{covariate}_1) + \ldots + \beta_n(\text{covariate}_n)$

Dependent Variable: LSM.

Independent Variable: NHHR.

Covariates: age, gender, race, education, marital status, poverty income ratio, BMI, smoking status, physical activity, HEI-2015, hypertension, and diabetes.

Supplementary Table 1 Associations between NHHR and CAP

	Model 1[a]		Model 2[b]		Model 3[c]	
	β (95% CI)	*p* value	β (95% CI)	*p* value	β (95% CI)	*p* value
Continuous	14.53 (13.03, 16.02)	<0.001	13.64 (12.14, 15.15)	<0.001	7.41 (6.07, 8.75)	<0.001
Categories						
T1	Reference		Reference		Reference	
T2	23.40 (18.60, 28.19)	<0.001	21.64 (16.94, 26.34)	<0.001	11.91 (7.83, 16.00)	<0.001
T3	49.91 (45.11, 54.70)	<0.001	46.44 (41.61, 51.27)	<0.001	25.83 (21.51, 30.16)	<0.001
p for trend		<0.001		<0.001		<0.001

Results are presented as β (95% CI) and *p* value, with *p* value <0.05 indicating the statistical significance of the association. NHHR is analyzed both as a continuous variable to assess the linear relationship and as a categorical variable (tertiles) to explore the trend. *p* values for trend tests indicates the significance of the linear trend across tertiles, with *p* for trend value <0.05 indicating a significant trend.

[a] Model 1: adjusted for no covariates.

[b] Model 2: adjusted for age, gender, and race.

[c] Model 3: adjusted for age, gender, race, education, marital status, poverty income ratio, BMI, smoking status, physical activity, HEI-2015,

hypertension, and diabetes.

Abbreviations: CAP, controlled attenuation parameter; CI, confidence interval; NHHR, non-high-density lipoprotein cholesterol to high-density lipoprotein cholesterol ratio; T, tertile

Supplementary Table 2 Associations between NHHR and LSM

	Model 1[a]		Model 2[b]		Model 3[c]	
	β (95% CI)	*p* value	β (95% CI)	*p* value	β (95% CI)	*p* value
Continuous	0.19 (0.07, 0.31)	0.001	0.18 (0.06, 0.31)	0.003	0.00 (-0.13, 0.12)	0.998
Categories						
T1	Reference		Reference		Reference	
T2	-0.05 (-0.43, 0.34)	0.818	-0.07 (-0.46, 0.31)	0.716	-0.35 (-0.73, 0.04)	0.076
T3	0.62 (0.23, 1.00)	0.002	0.58 (0.19, 0.98)	0.004	-0.03 (-0.44, 0.37)	0.867
p for trend		0.002		0.004		0.866

Results are presented as β (95% CI) and *p* value, with *p* value <0.05 indicating the statistical significance of the association. NHHR is analyzed both as a continuous variable to assess the linear relationship and as a categorical variable (tertiles) to explore the trend. *p* values for trend tests indicates the significance of the linear trend across tertiles, with *p* for trend value <0.05 indicating a significant trend.

[a] Model 1: adjusted for no covariates.

[b] Model 2: adjusted for age, gender, and race.

[c] Model 3: adjusted for age, gender, race, education, marital status, poverty income ratio, BMI, smoking status, physical activity, HEI-2015,

hypertension, and diabetes.

Abbreviations: CI, confidence interval; LSM, liver stiffness measurement; NHHR, non-high-density lipoprotein cholesterol to high-density lipoprotein cholesterol ratio; T, tertile

Supplementary Table 3 Threshold effect analysis of NHHR on CAP

Outcome	β (95% CI)	*p* value
One - line linear regression model [a]	7.41 (6.07, 8.75)	<0.001
Two - piecewise linear regression model [b]		
NHHR≤4	12.66 (10.65, 14.67)	<0.001
NHHR>4	-0.18 (-2.73, 2.38)	0.891
Log - likelihood ratio test [c]		<0.001

Results are presented as β (95% CI) and *p* value, with *p* value < 0.05 indicating the statistical significance of the association.

[a] One - line linear regression model assesses the association between NHHR and CAP using a single linear relationship across the entire range of NHHR values.

[b] Two - piecewise linear regression model assesses the association between NHHR and CAP with a potential threshold effect at a NHHR value of 4. The relationship is analyzed separately for NHHR values ≤4 and >4.

[c] Log - likelihood ratio test compares the one-line linear regression model with the two-piecewise linear regression model to determine if the two-piecewise linear regression model significantly improves the fit. A *p* value <0.05 indicates that the two-piecewise linear regression model provides a significantly better fit to the data compared to the one-line linear regression model.

Abbreviations: CAP, controlled attenuation parameter; CI, confidence interval; NHHR, non-high-density lipoprotein cholesterol to high-density lipoprotein cholesterol ratio

Supplementary Table 4 Threshold effect analysis of NHHR on LSM

Outcome	β (95% CI)	*p* value
One - line linear regression model [a]	0.00 (-0.13, 0.12)	0.998
Two - piecewise linear regression model [b]		
NHHR≤2.2	-0.37 (-0.87, 0.12)	0.142
NHHR>2.2	0.07 (-0.09, 0.22)	0.394
Log - likelihood ratio test [c]		0.128

Results are presented as β (95% CI) and *p* value, with *p* value < 0.05 indicating the statistical significance of the association.

[a] One - line linear regression model assesses the association between NHHR and CAP using a single linear relationship across the entire range of NHHR values.

[b] Two - piecewise linear regression model assesses the association between NHHR and CAP with a potential threshold effect at a NHHR value of 2.2. The relationship is analyzed separately for NHHR values ≤2.2 and >2.2.

[c] Log - likelihood ratio test compares the one-line linear regression model with the two-piecewise linear regression model to determine if the two-piecewise linear regression model significantly improves the fit. A *p* value <0.05 indicates that the two-piecewise linear regression model provides a significantly better fit to the data compared to the one-line linear regression model.

Abbreviations: CI, confidence interval; LSM, liver stiffness measurement; NHHR, non-high-density lipoprotein cholesterol to high-density lipoprotein cholesterol ratio

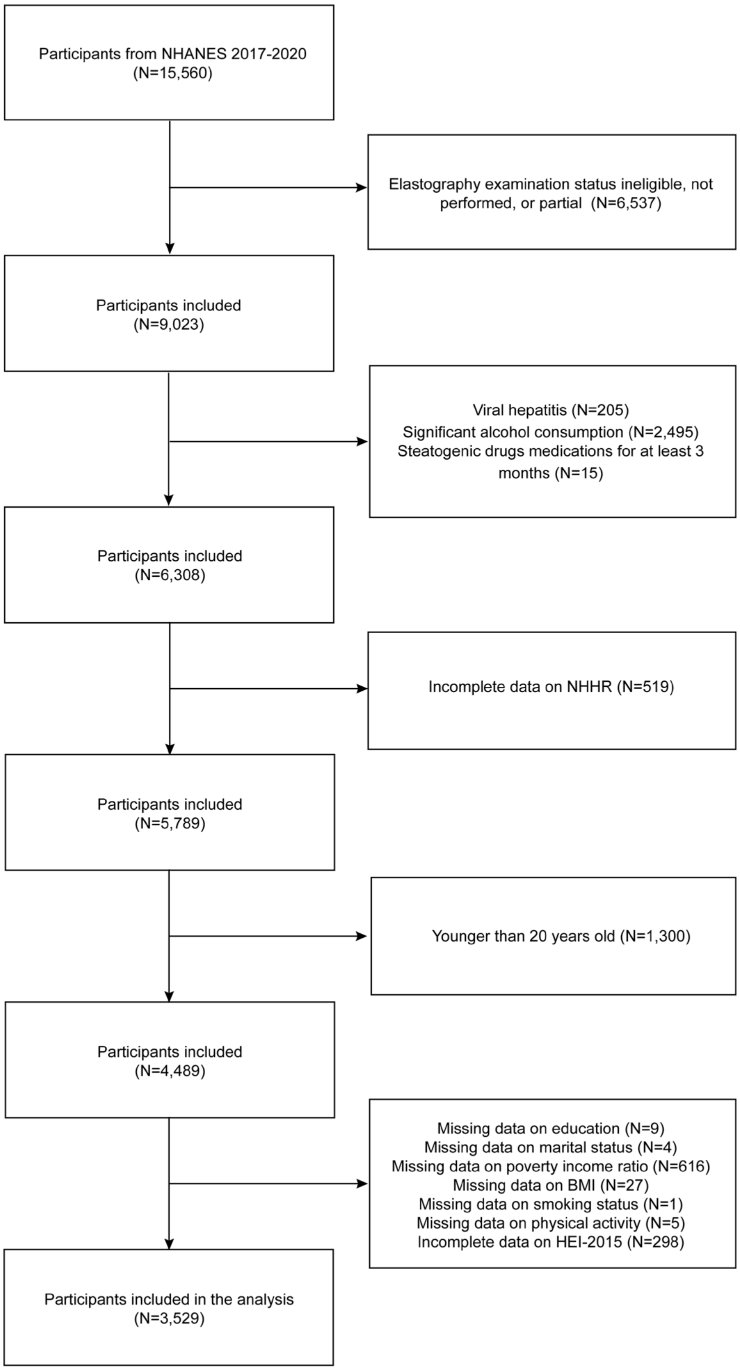

Supplementary Figure 1 Flow chart of participants selection

Abbreviations: BMI, body mass index; NHANES National Health and Nutrition Examination Survey; NHHR, non-high-density lipoprotein cholesterol to high-density lipoprotein cholesterol ratio; HEI-2015, Healthy Eating Index-2015

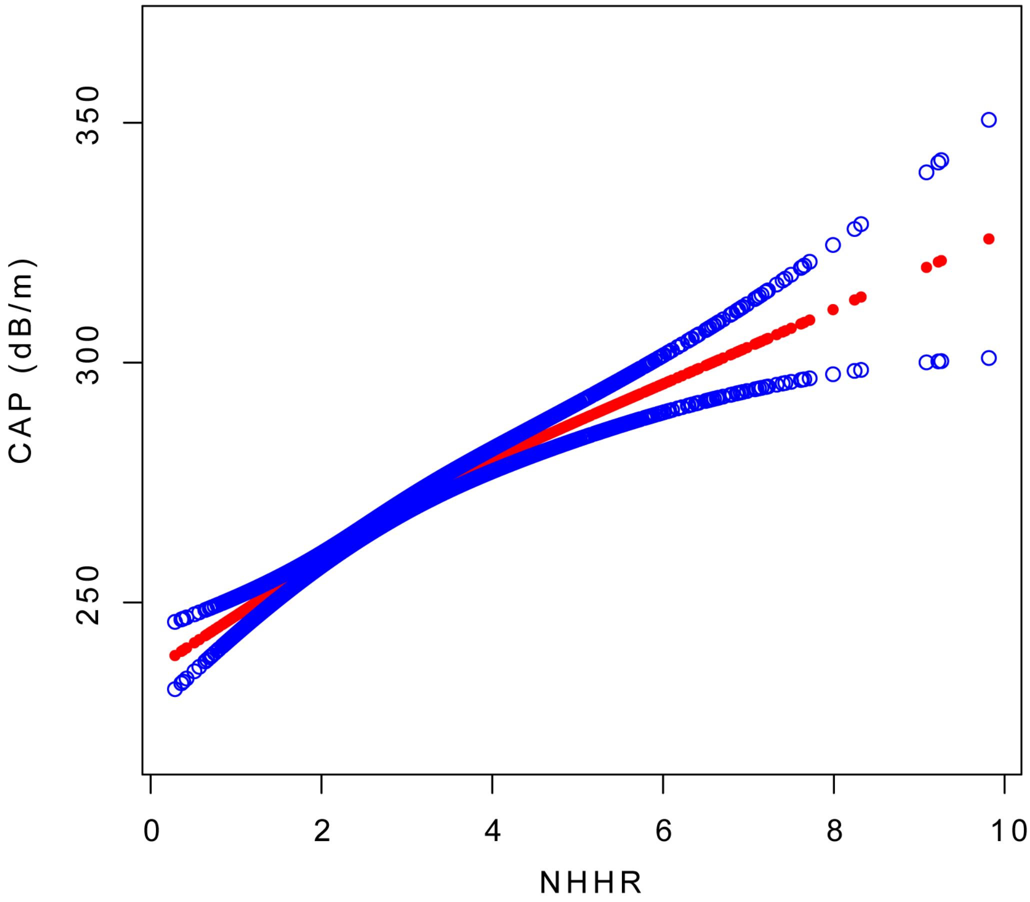

Supplementary Figure 2 The association between NHHR and CAP

The solid red line represents the smooth curve fitting between variables, with blue bands representing the 95% CI of the fitting.

Abbreviations: CAP, controlled attenuation parameter; NHHR, non-high-density lipoprotein cholesterol to high-density lipoprotein cholesterol ratio

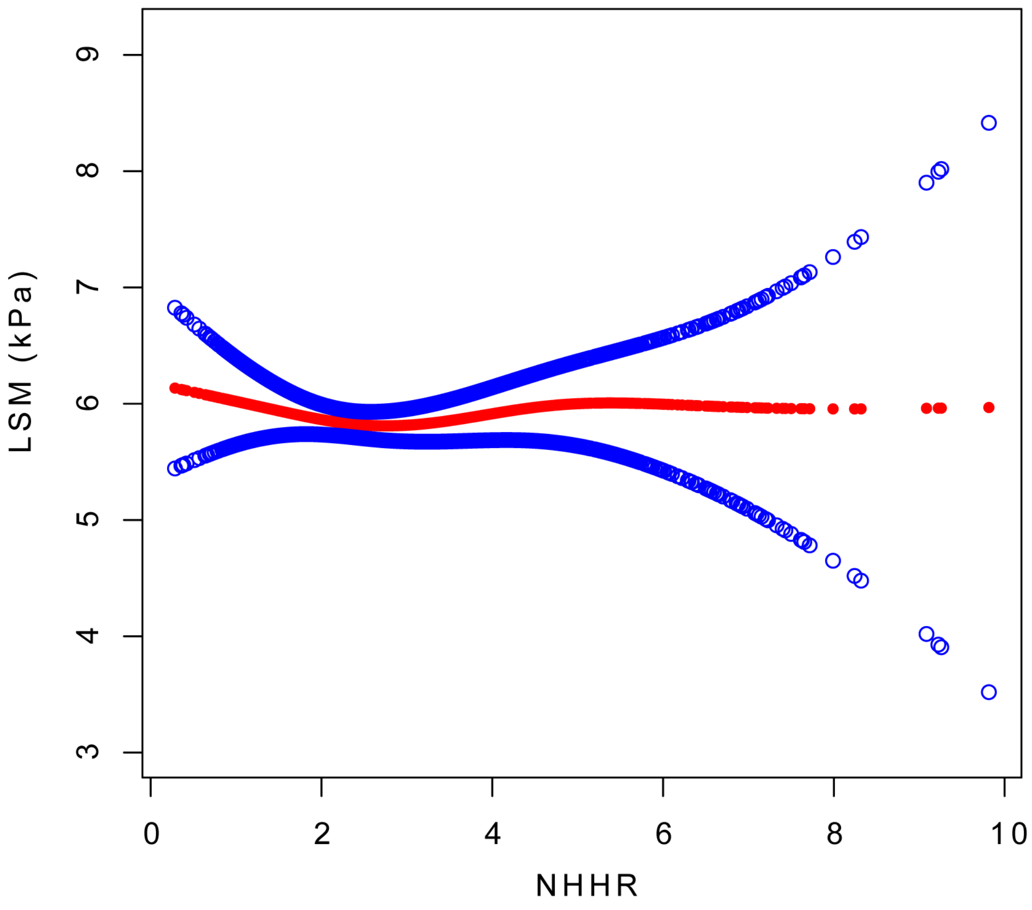

Supplementary Figure 3 The association between NHHR and LSM

The solid red line represents the smooth curve fitting between variables, with blue bands representing the 95% CI of the fitting.

Abbreviations: LSM, liver stiffness measurement; NHHR, non-high-density lipoprotein cholesterol to high-density lipoprotein cholesterol ratio
